# *In vitro* antitumor and immunomodulatory activities of 1,2,4-oxadiazole derivatives

**DOI:** 10.1016/j.bbrep.2025.101950

**Published:** 2025-02-10

**Authors:** Héverton Mendes Araújo, Gabriel Acácio de Moura, Yasmim Mendes Rocha, Cristian Vicson Pinheiro Gomes, Valentina Nascimento e Melo de Oliveira, Ronaldo Nascimento de Oliveira, Larissa Deadame de Figueiredo Nicolete, Emanuel Paula Magalhães, Ramon R.P.P.B. de Menezes, Roberto Nicolete

**Affiliations:** a Post-Graduate Program in Pharmaceutical Sciences (PPGCF), Federal University of Ceará (UFC), Fortaleza, CE, Brazil; b Fundação Oswaldo Cruz, Fiocruz, Fiocruz Ceará, Eusébio, CE, Brazil; c Instituto Federal de Educação Ciência e Tecnologia de Pernambuco, Campus Ipojuca, Ipojuca-PE, Brazil; d Department of Chemistry, Rural Federal University of Pernambuco, Recife-PE, Brazil; e Health Sciences Institute, University of International Integration of the Afro-Brazilian Lusophony (UNILAB), Redenção, CE, Brazil

**Keywords:** 1,2,4 oxadiazole, Macrophages, Polarization, Murine melanoma, Immunotherapy

## Abstract

Melanoma is the most aggressive and lethal type of skin cancer, responsible for approximately 60,000 deaths annually. The main strategy for treating melanoma is surgery to completely remove the lesion and its margins. However, for more advanced cases with a high recurrence rate, the preferred approach is to combine chemotherapy with immunotherapy treatments. Tumor-associated macrophages (TAMs) are the most abundant leukocytes in solid tumors. Current immunotherapy approaches target TAMs by inhibiting pro-tumoral TAMs and activating anti-tumoral TAMs, repolarizing them to the M1 phenotype. The antitumor and immunomodulatory activities of molecules derived from 1,2,4-oxadiazole, as demonstrated in the literature, highlight the potential of this class as a source of promising candidates for therapeutic applications. Thus, the present study aims to evaluate the antitumor and immunomodulatory effects of the synthetic derivative 1,2,4-oxadiazole, N-cyclohexyl-3-(3-methylphenyl)-1,2,4-oxadiazole-5-amine (1,2,4-oxadiazole derivative 2), in melanoma cells and murine Bone Marrow-Derived Macrophages (BMDMs). Cytotoxicity in B16–F10 and BMDMs cells was assessed using the (3-(4,5-dimethylthiazol-2-yl)-2,5-diphenyltetrazolium bromide) MTT method. 1,2,4-oxadiazole derivative 2 exhibited antiproliferative effects on both cell lines, being 2.6 times more selective for B16–F10. Necrosis was identified as the active induced death pathway. BMDMs isolated and exposed to 1,2,4-oxadiazole derivative 2 polarize to the M1 phenotype and induce TNF-α at a concentration of 64.34 μM. Exposure to melanoma murine supernatants also promotes M1 polarization. Supernatants containing traces of 1,2,4-oxadiazole derivative 2 (Supernatants B, C, and D) increased the percentage of M1 cells compared to Supernatant A, as well as elevated levels of nitrite, TNF-α, and IL-12. 1,2,4-oxadiazole derivative 2 combined with Supernatant A and 1,2,4-oxadiazole derivative 2 combined with LPS also resulted in higher M1 polarization, suggesting a synergistic effect on M1 polarization and TNF-α production. Our findings underscore the significance of the 1,2,4-oxadiazole compound class and highlight the potential of 1,2,4-oxadiazole derivative 2 as an antitumoral and immunotherapeutic agent.

## Highlights

• 1,2,4-oxadiazole derivative 2 induces cell death via necrosis.
• 1,2,4-oxadiazole derivative 2 polarizes BMDMs towards the M1 phenotype.
• Murine melanoma supernatants induce production of NO, TNF-α, and IL-12.
• 1,2,4-xadiazole derivative 2 displays synergistic effect on the polarization induced by LPS and supernatants.

## 1 Introduction

Melanoma is a skin tumor from melanocytes, pigment-producing cells distributed throughout the body [1]. It occurs primarily because of exposure to ultraviolet radiation, which directly damages Deoxyribonucleic acid (DNA) [2]. Like other carcinogenesis processes, successive and cumulative somatic mutations affect genes involved in proliferation, growth, metabolism, cell cycle, and cell death, conferring these cells advantages over other healthy cells. Keratinocytes control escape favors melanocyte formation and dysplastic nevi. Also, immune system inhibitory mechanisms escape culminating in transforming benign tumors into malignant ones with phenotypic alterations [3]. According to the World Health Organization (WHO), in 2022 there were 330,000 new cases of melanoma skin cancer, with nearly 60,000 deaths, underscoring the significant global impact of this disease [4].

Some treatment approaches can be employed for melanoma conditioned by type and disease stage. The primary strategy is surgery with complete lesion and margin removal. Patients in stages II-IV have a 30–90 % risk of recurrence causing the administration of immunotherapeutic agents such as dabrafenib, trametinib, and nivolumab. A novel therapeutic strategy for advanced locoregional melanomas using anti-programmed cell death protein 1 (PD-1) and anti-cytotoxic T-lymphocyte antigen 4 (CTLA-4) immunotherapy has shown efficacy as a preoperative treatment to enhance cure rates and shorten the duration of systemic therapy [5].

Aligned with the current use of immunotherapy, there is a critical need to explore novel multivalent molecules with maximum tumor-specific toxic effects and minimal adverse effects. These molecules should also stimulate the immune system to enhance its effectiveness in destroying cancer cells. When combined within a single molecule, such pharmacological properties offer promising prospects for antitumor treatments, whether as an adjuvant or primary therapy [6].

The beginning of melanoma development invariably triggers inflammatory processes. Macrophages, in higher numbers, and other leukocytes are recruited to eliminate malignant cells [7]. They are important effector cells of the innate immune system and regulators of the adaptive immune system, involved in tasks such as pathogen and tumor cell recognition and destruction, antigen presentation, tissue damage repair, cell signaling, and modulation of inflammatory processes. A remarkable characteristic is cellular plasticity [8].

Pro-inflammatory factors such as Lipopolysaccharides (LPS), Interferon-gamma (IFN-γ), Tumor Necrosis Factor-Alpha (TNF-α), and Toll-like receptor (TLR) ligands activate the classical pathway in macrophages, leading to the M1 phenotype. This activation results in the production of Interleukins (IL) 1, 12 and 23, TNF-α, and chemokine Interferon-γ inducible protein 10 kDa (CXCL10), as well as reactive oxygen and nitrogen species (ROS and RNS), and nitric oxide (NO). Additionally, it enhances the expression of Major Histocompatibility Complex (MHC) molecules, which are essential proteins for the immune system, binding with a variety of antigens and presenting them to lymphocytes, facilitating antigen recognition and immune system activation. These characteristics endow M1 macrophages with high cytotoxic activity, making them efficient pathogens and cancer cell killers. Alternative activation induces the M2 phenotype in response to IL-4, IL-10, IL-13, IL-33, and Transforming Growth Factor-Beta (TGF-β). M2 macrophages are important for wound healing, tissue remodeling, the production of anti-inflammatory cytokines, and immunosuppression [9]. The M2 phenotype is associated with activation of Phosphoinositide 3-kinase/protein kinase B (PI3K/AKT), Janus kinase-signal transducer and activator of transcription 3 or 6 (JAK/STAT6 or STAT3), and TGF-β/SMAD-dependent pathways. The c-Jun N-terminal kinase (JNK), p38, Nuclear Factor kappa B (NF-κB) p65, JAK/STAT1, and NOTCH pathways are associated with the M1 phenotype [10].

Tumor-associated macrophages (TAMs) presence in the tumor microenvironment (TME) is generally associated with therapeutic resistance. However, new studies have shown a counterpoint to this view and approached these cells not only as prognostic markers but also as potential therapeutic targets. Considering that they can perform opposite roles in the TME, TAMs represent an important target for exploration. Current strategies targeting TAMs are based on two approaches: inhibiting pro-tumoral TAMs, which involves inhibiting recruitment and elimination, and activating anti-tumoral TAMs, which involves repolarizing M2 macrophages into M1 [11].

Oxadiazole is a class of chemical compounds characterized by a heterocycle consisting of two carbon atoms, two nitrogen atoms, one oxygen atom, and two double bonds. Variations in the position of these atoms yield four isomers: 1,2,3-oxadiazole, 1,2,4-oxadiazole, 1,3,4-oxadiazole, and 1,2,5-oxadiazole [12].

The 1,2,4-oxadiazole ring is highly regarded for its versatility and has been extensively investigated for therapeutic applications [13,14]. They are fundamental pharmacophoric groups that confer stability in aqueous media [15] and can be used as bioisosteres of amides and esters [16]. It has been previously reported that compounds based on 1,2,4-oxadiazole can activate immune checkpoints and suppress the proliferation of diverse tumor cell, suggesting their potential application as cancer treatment [17].

Regarding the anti-tumor activity, literature demonstrates that compounds derived from the 1,2,4-oxadiazole ring are capable of inducing cell death in a wide range of tumor cell lines, including breast (MCF-7, MDA-MB-231, HBL100, T47D, PyMT-B01, and 4TI), prostate (PC3), colon (SW111, HT29, WiDr, and HCT116), skin (Du145, B16–F10, and SKMEL103), lung (A549, SW1573, Lewis, NCIH460, and NCIH226), leukemia (MV411, MOLM13, THP-1, OCI-AML3, NB4, HL, CMK, CCRF-CEM, HL69, SR, and others), through various mechanisms such as apoptosis, necrosis, telomerase inhibition, tubulin polymerization inhibition, cell cycle arrest, and alteration of mitochondrial membrane potential. Considering their immune system modulating activity, derivatives of the 1,2,4-oxadiazole ring are attributed to reducing and increasing leukocyte infiltrates in edemas, enhancing or reducing NO production, reducing pro-inflammatory cytokines such as IL-1β, IL-6, IL-8, TNF-α, reducing NF-кB pathway activation, and exhibiting anti-inflammatory activities by inhibiting cyclooxygenase (COX) enzymes [[18], [19], [20], [21]].

Our previous studies about 1,2,4-oxadiazole derivatives have shown cytotoxic activity in prostate, colon, central nervous system, stomach, and lung tumor cell lines [22,23]. This has motivated us to delve deeper into this anti-tumor mechanism and explored the immunomodulatory activity of these compounds. We hypothesized that the synthetic 1,2,4-oxadiazole derivative 2, N-cyclohexyl-3-(3-methylphenyl)-1,2,4-oxadiazole-5-amine, also exhibits anti-tumor effects in melanoma cells and immunomodulatory activity in murine Bone Marrow-Derived Macrophages (BMDMs) by modulating specific pathways involved in tumor proliferation and immune responses. Therefore, the present study aims to evaluate the anti-tumor and immunomodulatory activities of the 1,2,4-oxadiazole derivative 2 by employing different *in vitro* cellular experiments.

## 2 Materials and methods

### 2.1 Chemicals

**Fig. 1:**
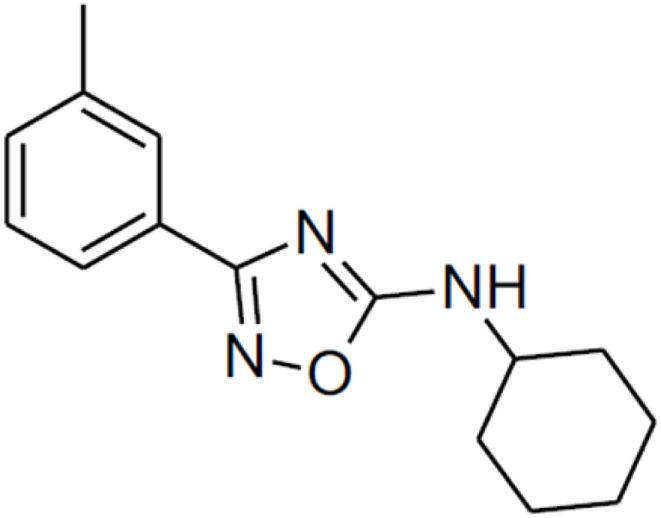
1,2,4-oxadiazole derivative 2 chemical structure.

1,2,4-oxadiazole derivative N-cyclohexyl-3-(3-methylphenyl)-1,2,4-oxadiazol-5-amine, was synthesized from a solution of arylamidoximes and dicyclohexylcarbodiimide (DCC 1) in DMF for 10 min under microwave in the Laboratory of Bioactive Compound Synthesis at the Federal Rural University of Pernambuco, as detailed for [22]. We refer to this compound as 1,2,4-oxadiazole derivative 2 to maintain the same nomenclature in previous published studies. The chemical structure of 1,2,4-oxadiazole derivative 2 is present in Fig. 1.

### 2.2 Tumor cell line

Murine melanoma cell line B16–F10 was donated by the Experimental Oncology Laboratory of Federal University of Ceará (UFC), Fortaleza-CE, Brazil. The cells were cultured in RPMI 1640 medium (Gibco, Carlsbad, CA) supplemented with 10 % fetal bovine serum (FBS) (LGC Biotecnologia Ltda.) and 1 % penicillin-streptomycin solution (200 U/mL penicillin and 130 mg/L streptomycin) (LGC Biotecnologia Ltda.). All cultures were grown in sterile plastic flasks (KASVI) and maintained in incubators at 37 °C ± 0.3 °C in a 5 % Carbon Dioxide (CO_2_) atmosphere.

### 2.3 Animals and Bone Marrow-Derived Macrophages (BMDMs)

Female C57BL/6 isogenic mice, aged 6–8 weeks and weighing 19–22 g, were acquired from the Bioterium of the Center for Drug Research and Development (NPDM) at the UFC and housed in their facility. They were conditioned to 12/12-h light/dark cycle and an ambient temperature of around 23 °C, with food and water provided ad libitum. The entire protocol was conducted and approved in accordance with the recommendations of the NPDM Animal Ethics Committee under number 9676290319.

Macrophages were obtained from mice bone marrow. After euthanasia by administering a lethal dose of the anesthetics Ketamine and Xylazine, immature cell-containing bone marrow was extracted from the femurs and tibias of the animals, and the material was brought to the laboratory for MDMO differentiation. The cell pellet obtained was cultured in macrophage differentiation medium composed high-glucose DMEM (Gibco, Carlsbad, CA) supplemented with 10 % FBS (LGC Biotecnologia Ltda.), 1 % penicillin-streptomycin solution (200 U/mL penicillin and 130 mg/L streptomycin) (LGC Biotecnologia Ltda.), and 10 ng/mL Macrophage Colony-Stimulating Factor (CSF-M) (Sigma-Aldrich Brasil Ltda.). After seven days, cellular differentiation was completed [24], yielding between 2.5 × 10^6^ and 3.5 × 10^6^ mature BMDMs.

### 2.4 Cytotoxicity assay

1,2,4-oxadiazole derivative 2 was tested *in vitro* for its ability to inhibit cell proliferation using the MTT assay. Briefly, B16–F10 and BMDMs were plated at a density of 1 × 10^5^ cells/well in 96-well plates and treated with various 1,2,4-oxadiazole derivative 2 concentrations (3.125–400 μM) and 0.4 % of vehicle group dimethyl sulfoxide (DMSO). All plates were incubated for 72 h at 37 °C ± 0.3 °C in a 5 % CO_2_ atmosphere. After that, MTT solution (2.5 mg/mL) was added to the wells and incubated for 3 h. Then, plates were centrifuged, supernatant removed, and formazan crystals were solubilized with DMSO. Finally, analysis was performed using a spectrophotometer at 570 nm on a MULTISKAN FC equipment (Thermo Scientific, USA) [25]. The inhibition ratio (IR) of cell proliferation was calculated by the following formula: IR (%) = (1-experiment group OD/control group OD) × 100.

### 2.5 Flow cytometry assays

#### 2.5.1 Cell death assay

Cell death was evaluated by staining B16–F10 cells with 7-Aminoactinomycin D (7-AAD) and Annexin V conjugated with the fluorochrome Phycoerythrin (AxPE) according to the manufacturer's instructions (eBioscience, SanDiego, CA, USA) followed by analysis using flow cytometry. Briefly, B16–F10 cells were planted at a density of 1 × 10^6^ cells/well in 12-well plates and treated with fixed 1,2,4-oxadiazole derivative 2 concentrations 50.99, 76.48, 101.98 μM corresponding to half-maximal inhibitory concentration (IC_50_) 1.5xIC_50,_ 2xIC_50_ calculated from cytotoxicity assay. All plates were incubated for 48 h at 37 °C ± 0.3 °C in a 5 % CO_2_ atmosphere. Following incubation, supernatants from the negative control (referred to as Supernatant A) and from the concentrations of 50.99, 76.48, and 101.98 μM (referred to as Supernatants B, C, and D respectively) were collected and stored for subsequent use in BMDM stimulation assays. Subsequently, the cells were collected, labeled with 7-AAD and AxPE and incubated for 15 min, protected from light at room temperature. Finally, the groups were analyzed on the FACSCalibur flow cytometer (BD Biosciences, NJ, USA) [26]. Each sample was analyzed for a minimum of 10^4^ events to determine the percentage of cells labeled with each fluorochrome, cells that were not labeled, or cells double-labeled with 7-AAD and AxPE.

#### 2.5.2 BMDMs identification

The maturation process and phenotype of BMDMs were determined based on Forward Scatter (FSC) and Side Scatter (SSC) characteristics, as well as surface marker anti-F4/80-PE (BioLegend, Inc., San Diego, CA, USA). Cell population was initially selected based on size and cytoplasmic complexity on FSC x SSC graphic (gate 1). The confirmation that the selected population at gate 1 comprised mature macrophages, confirming cellular differentiation, was achieved by constructing a new FSC vs anti-F4/80-PE graphic correlating cell size with the positivity of the anti-F4/80 marker (gate 2). The characterization of the cell population as mature BMDMs was confirmed by positive staining for anti-F4/80-PE [27].

#### 2.5.3 BMDMs phenotype identification assay after exposure to 1,2,4-oxadiazole derivative 2, LPS and murine melanoma supernatants

BMDMs phenotype alterations due exposure to 1,2,4-oxadiazole derivative 2, LPS and melanoma murine supernatants were determinate based on surface marker expression with anti-F4/80-PE, anti-CD11b-FITC, and anti-CD206-Alexa Fluor® 647 (BioLegend, Inc., San Diego, CA, USA). After identifying mature BMDMs, they were differentiated into M1 and M2 phenotypes using the following criteria: M1 phenotype macrophages displayed positive markers for anti-F4/80-PE and anti-CD11b-FITC, while M2 phenotype macrophages were positive for anti-F4/80-PE and anti-CD206-Alexa Fluor® 647 [27].

BMDMs were plated at a density of 1 × 10^6^ cells/well in 12-well plates and stimulated with 1,2,4-oxadiazole derivative 2 concentrations 33.17 and 64.34 μM, corresponding to 0.25IC_50_ and 0.5IC_50_ calculated from cytotoxicity assay, LPS 500 ng/mL, Supernatants A, B, C, and D and DMEM medium as negative control. All groups were incubated for 48 h and maintained in an atmosphere containing 5 % CO2 at 37 °C. After incubation, the cells were disaggregated from the surface of the plate using mechanical force and labeled with the aforementioned markers for 15 min protected from light. The labeled cells were then analyzed in FACSCALIBUR (BD Biosciences, NJ, USA). Supernatants from all groups were collected for nitrite, TNF-α and IL-12 quantification.

### 2.6 Nitrites measurement

Cell supernatants collected from BMDMs phenotype identification assay were submitted to nitrite (NO_2_^−^) dosage using the colorimetric GRIESS method according to the manufacturer's instructions (Promega Corporation, WI, USA). The standard curve was prepared using a standard solution of 100 μM NaNO_2_ included in the kit, followed by serial dilutions (50; 25; 12.5; 6.25; 3.125; 1.56 μM). Initially, 50 μL of the standards and supernatants were added to wells 96-well plate, followed by the addition of 50 μL of sulfanilamide solution and incubation for 10 min at room temperature, protected from light. In the next step, 50 μL of (1-naphthylethylenediamine) Dihydrochloride (NED) solution were added to each well and incubated again for 10 min at room temperature, protected from light. After incubation, the absorbance was measured at 530 nm using the MULTISKAN FC spectrophotometer (Thermo Scientific, USA) [28].

### 2.7 TNF-α and IL-12 measurement

Cell supernatants collected from BMDMs phenotype identification assay were submitted to TNF-α and IL-12 determination by Enzyme-Linked Immunosorbent Assay (ELISA) according to the manufacturer's instructions (MyBioSource, San Diego, CA, USA). The standard curve was prepared using a standard solution of 1000 pg/mL followed by serial dilutions (500; 250; 125; 62.5; 31.25; 15.6 pg/mL). Initially, the standards and supernatants were added to wells coated with antibodies against the cytokines and incubated for 90 min at 37 °C. Then, 100 μL of biotinylated detection antibody was added and incubated for 60 min at 37 °C. After incubation, the supernatant was aspirated, and the wells were washed with a streptavidin-HRP solution for 30 min at 37 °C. The wells were then aspirated and washed with a substrate and chromogen solution, followed by a 15-min incubation at 37 °C. Finally, the absorbance was measured at 450 nm using the MULTISKAN FC spectrophotometer (Thermo Scientific, USA) [29,30]."

### 2.8 Statistical analysis

All statistical analyses were performed with GraphPad Prism® software version 8.0. Data were expressed as mean ± standard error of the mean (SEM). Comparison between groups were performed using One-Way ANOVA and Two-Way ANOVA followed by Dunnett's or Bonferroni's post-test. For cytotoxicity tests, p < 0.05 was considered as statistically significant, and for flow cytometry, nitrites dosage, and TNF-α and IL-12, p < 0.001 was considered as statistically significant.

## 3 Results

### 3.1 1,2,4-Oxadiazole derivative 2 exhibits antiproliferative effects on the B16–F10 cell line

**Fig. 2:**
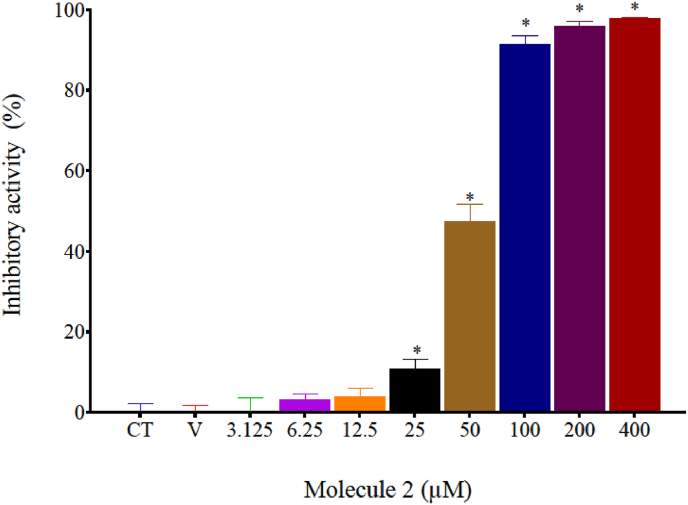
1,2,4-oxadiazole derivative 2 inhibits B16–F10 growth in a dose-dependent manner after 72h exposure. DMSO was used as Vehicle (V). Data were expressed as mean percentage of growth inhibition ± standard error mean (SEM) and evaluated by one-way ANOVA with Dunnet's post-test ∗p < 0.05 compared to control group (CT).

To evaluate if 1,2,4-oxadiazole derivative 2 exhibits cytotoxic activity in murine melanoma, we conducted *in vitro* assays on the B16–F10 cell line using the MTT colorimetric method as shown in Fig. 2. 1,2,4-oxadiazole derivative 2 inhibited the growth of B16–F10 cells in a dose-dependent manner at concentrations above 50 μM. The IC_50_ value was 50.99 ± 2.94 μM. No growth inhibition was observed with the DMSO vehicle control at a tested concentration of 0.4 %.

### 3.2 1,2,4-Oxadiazole derivative 2 induces cell death via necrosis

**Fig. 3:**
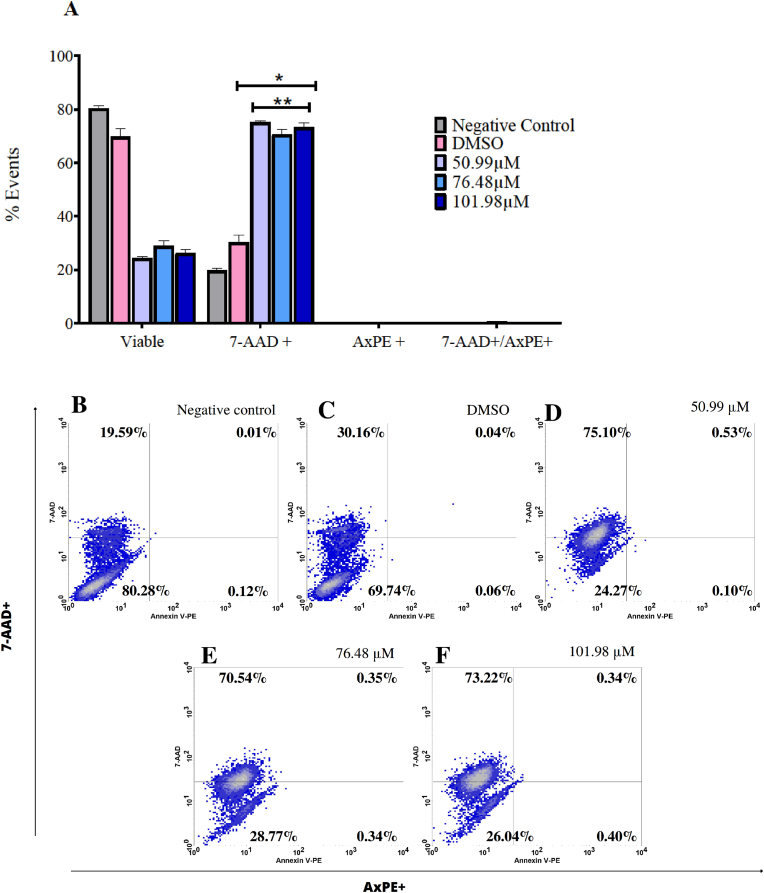
1,2,4-oxadiazole derivative 2 induces cell death in B16F10 via necrosis after 48 h of exposure. (A) Column graph indicating the percentage of viable cells not labeled, labeled by 7-AAD and/or labeled by AxPE. Data were expressed as percentage of events ± standard error mean (SEM) and evaluated by two-way ANOVA with Bonferroni post-test ∗p < 0.001 compared to negative control group. (B–F). Dotplot graph showing the behavior and marking percentages of each group.

Once known cytotoxic activity and IC_50_ value of 1,2,4-oxadiazole derivative 2 in B16–F10 cells, we used flow cytometry to evaluate the cell death pathway involved, distinguishing between apoptosis and necrosis, using the markers Annexin V-PE (AxPE) and 7-AAD as shown in Fig. 3. The assay was conducted after 48 h of exposure to 1,2,4-oxadiazole derivative 2 at concentrations of 50.99 μM (IC_50_), 76.48 μM (1.5 x IC_50_), and 101.98 μM (2 x IC_50_). We observed a significant increase in the percentage of cells labeled with 7-AAD at all three concentrations, with values of 75.10 %, 70.54 %, and 73.22 % respectively, compared to the negative control. These values did not significantly differ from each other. We also observed slight increase in percentages in vehicle group. Additionally, no significant differences were observed between the groups marked solely by AxPE or by both 7-AAD and AxPE.

### 3.3 1,2,4-Oxadiazole derivative 2 maintains the viability of BMDMs

**Fig. 4:**
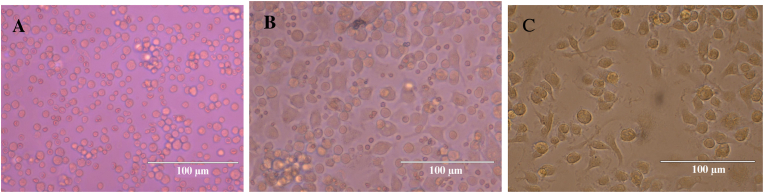
Differentiation of immature bone marrow cells into mature macrophages observed under optical microscopy 400x, scale bar = 100 μm. (A, B and C) first, fourth and seventh day respectively.

**Fig. 5:**
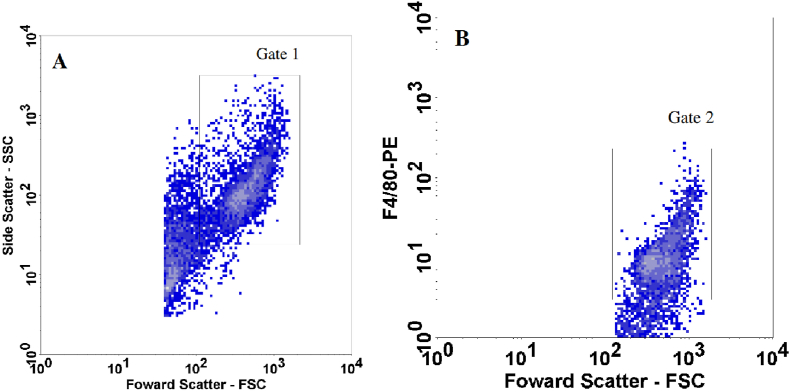
Dotplot graphs at the end BMDMs differentiation process. Gate 1 contains the analyzed cell population and Gate 2 confirms the differentiation of immature bone marrow cells into mature macrophages by positive staining for F4/80.

BMDMs were the final product of the collection and differentiation steps of immature cells from the bone marrow of C57BL/6 mice. Confirmation was achieved by observing the cells under optical microscopy and analyzing their size, complexity, and labeling with anti-F4/80-PE by flow cytometry. Fig. 4A depicts immature cells on the day of collection, showing populations with varying volume, homogeneous cytoplasm, rounded morphology, and presence of red blood cells. By Fig. 4B, on the fourth day of differentiation, transitions due to this process become evident. Cells show reduced volume variation, loss of cytoplasmic homogeneity, and some cells adopt oval morphology with cytoplasmic projections, and decrease in the number of red blood cells. Fig. 4C corresponds to the seventh and final day of the differentiation process, illustrating cells with small and similar volumes, a homogeneous population, heterogeneous cytoplasm, fusiform morphology with numerous cytoplasmic projections, spread-out growth, and an absence of red blood cells. In Fig. 5B, the evaluated cell population is depicted. The FSC vs F4/80-PE plot shows positive labeling for F4/80, confirming successful differentiation.

**Fig. 6:**
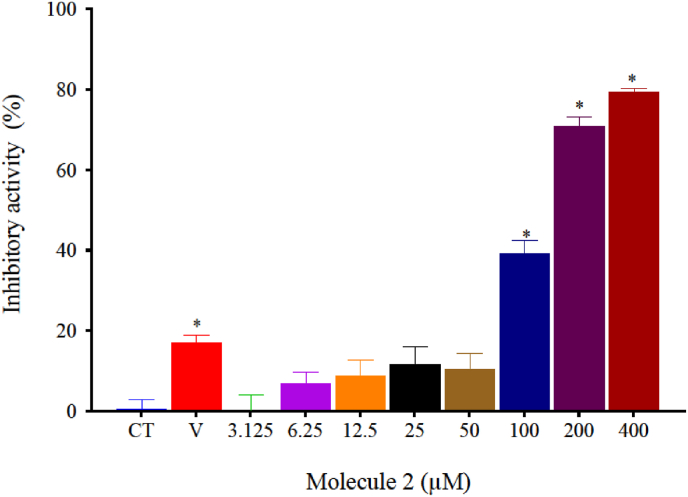
1,2,4-oxadiazole derivative 2 inhibits BMDMs growth in a dose-dependent manner after 72h exposure. DMSO was used as Vehicle (V). Data were expressed as mean percentage of growth inhibition ± standard error mean (SEM) and evaluated by one-way ANOVA with Dunnet's post-test ∗p < 0.05 compared to control group (CT).

The possible cytotoxic activity of 1,2,4-oxadiazole derivative 2 in BMDMs was also evaluated using MTT colorimetric method. As presented in Fig. 6, Fig. 1, Fig. 2, Fig. 4-oxadiazole derivative 2 inhibited macrophages growth in a dose-dependent manner at concentrations above 100 μM, the IC_50_ calculated was 132.7 μM (116.7–151.0 μM). The higher concentration tested for the vehicle (DMSO) was 0.4 % v/v per well. This group showed a slight reduction in cell viability. However, it can be inferred that the predominant cytotoxic effect is attributed to 1,2,4-oxadiazole derivative 2, given the more than 60 percentage point difference in inhibition between the vehicle and 1,2,4-oxadiazole derivative 2 at its highest concentration. Once we obtained the IC_50_ values for both cell lines, the selectivity index (SI) was calculated by dividing the IC_50_ values: B16–F10/BMDMs. The 1,2,4-oxadiazole derivative 2 was 2.6 times more selective for the tumor cell line than BMDMs.

### 3.4 1,2,4-Oxadiazole derivative 2 polarizes BMDMs towards the M1 phenotype and induces TNF-α production

**Fig. 7:**
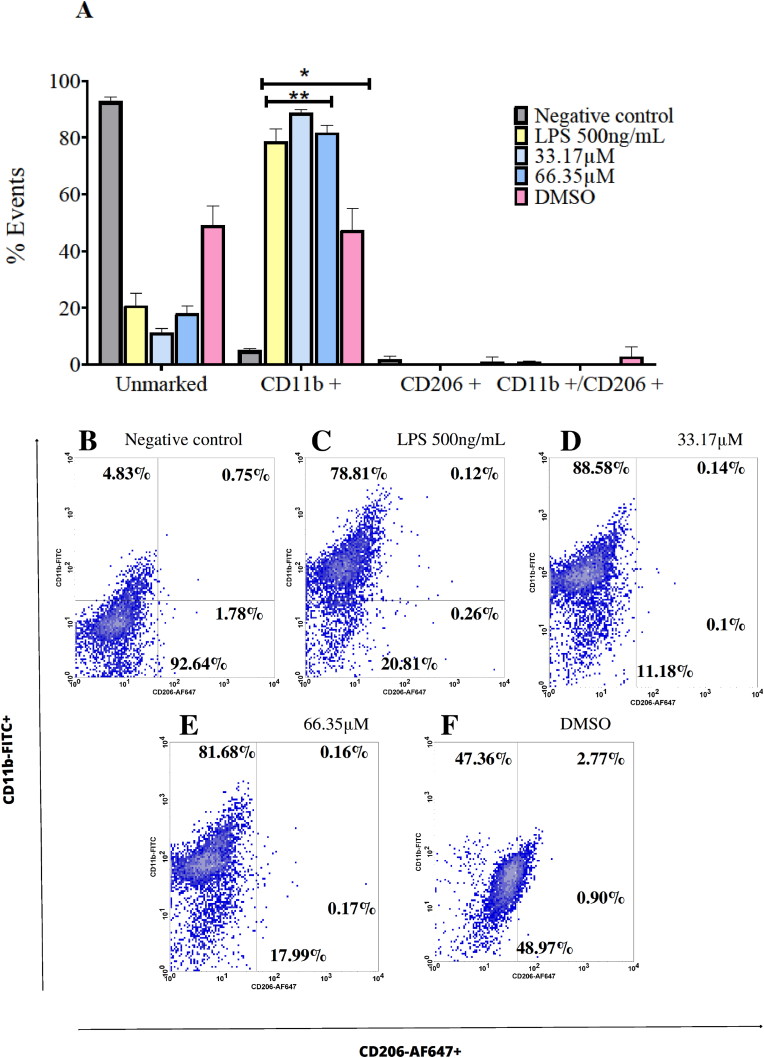
1,2,4-oxadiazole derivative 2 polarized BMDMs to M1 phenotype after 48h exposure. (A) Column graph indicating the percentage of unmarked cells, CD11b+, CD206+ and CD11b+/CD206+. Unstimulated cells acted as a negative control (M0). Data were expressed as percentage of events ± SEM and evaluated by two-way ANOVA with Bonferroni post-test ∗p < 0.001; compared to negative control group; ∗∗p < 0.001 compared to vehicle. (B–F). Dotplot graph showing the behavior and marking percentages of each group.

Using flow cytometry and markers anti-F4/80-PE, anti-CD11b-FITC, and anti-CD206-Alexa Fluor® 647, BMDMs were evaluated for their activation and subsequent acquired phenotypic alteration after exposure to 1,2,4-oxadiazole derivative 2 33.17 μM and 66.35 μM and LPS 500 ng/mL. All data are present in Fig. 7. It was observed high CD11b marker positivity in BMDMs exposed to LPS (78.81 %). Similarly, the two concentrations of 1,2,4-oxadiazole derivative 2 also exhibited significant CD11b positivity (88.58 % and 81.68 %). The vehicle group showed insignificant positivity for CD11b compared to LPS in 1,2,4-oxadiazole derivative 2.

**Fig. 8:**
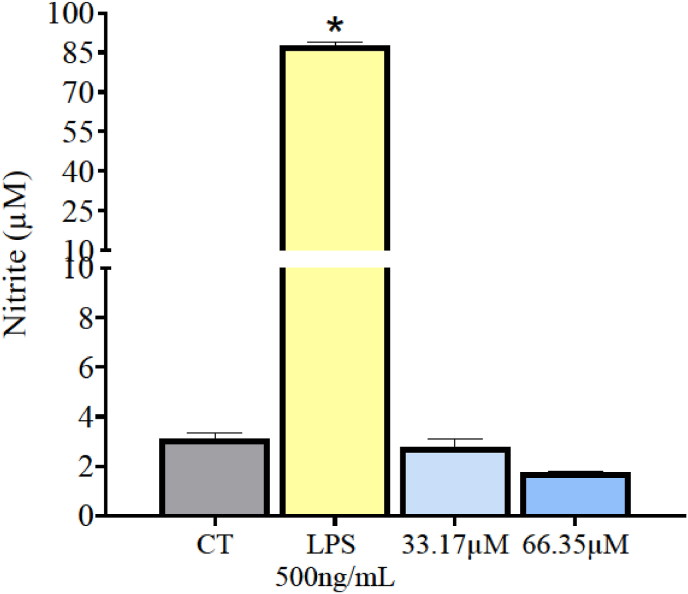
Dosage of the M1 marker TNF-α in BMDMs supernatants after 48h of exposure to 1,2,4-oxadiazole derivative 2 and LPS. Data were expressed as mean ± SEM and evaluated by one-way ANOVA with Dunnet's post-test ∗p < 0.001 compared CT. ND = Not detected.

TNF-α levels were measured in the supernatants of the LPS and 1,2,4-oxadiazole derivative 2 groups to assess activation via the classical pathway using ELISA assay. The results are shown in Fig. 8. The LPS-stimulated group produced high levels of these pro-inflammatory cytokine: 806.30 pg/mL. In comparison, the 1,2,4-oxadiazole derivative 2 stimulated groups displayed 102.75 pg/mL of TNF-α only at the higher concentration tested.

### 3.5 Murine melanoma supernatants polarize BMDMs towards the M1 phenotype and induce production of NO, TNF-α, and IL-12

BMDMs were evaluated for their phenotypic alteration after exposure to murine melanoma supernatants A, B, C and D. All samples likely expected to contain some DAMPs, as they were collected from the cell death identification assay and some percentage of cell death was identified in all groups. Additionally, it is also expected that the supernatants contain undetermined amounts of FDTs produced by B16–F10 cells. Supernatant A, collected from the negative control group, contained no traces of 1,2,4-oxadiazole derivative 2, as only complete culture medium was used. Supernatants B, C, and D were exposed to increasing concentrations of 1,2,4-oxadiazole derivative 2 and likely contained some amount of it.

**Fig. 9:**
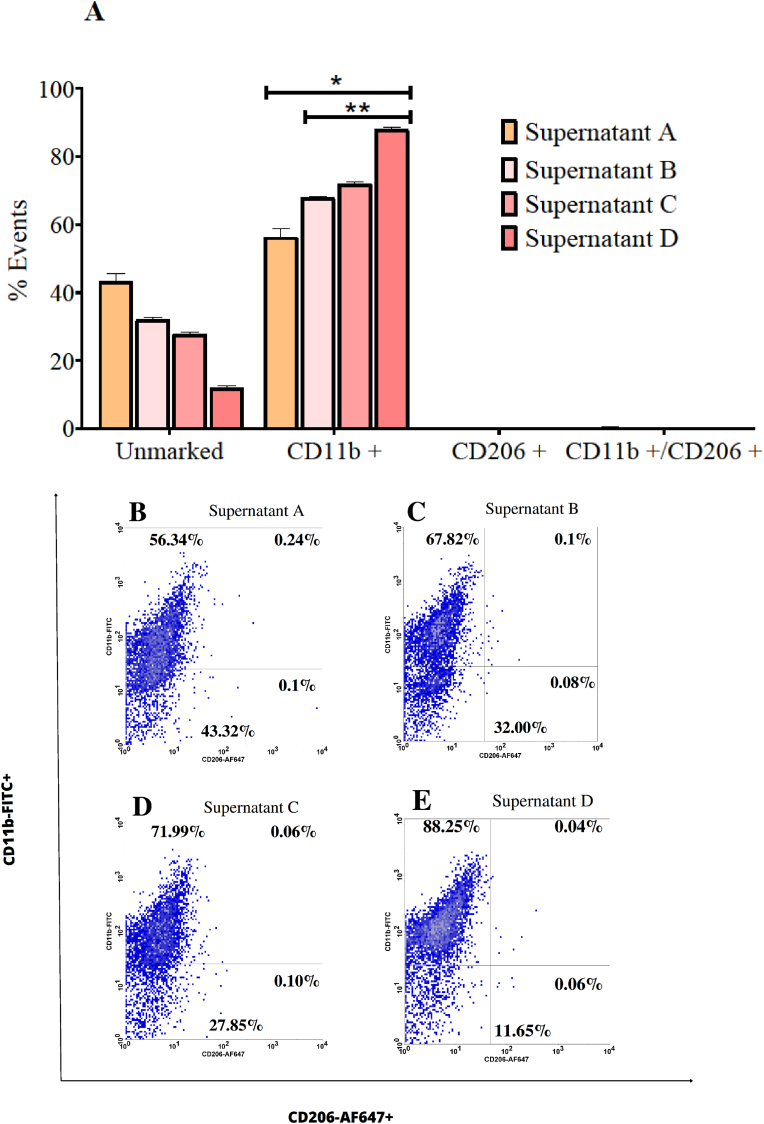
Murine melanoma supernatants polarized BMDMs to M1 phenotype after 48h exposure. (A) Column graph indicating the percentage of unmarked cells, CD11b+, CD206+ and CD11b+/CD206+. Unstimulated cells acted as a negative control (M0). Data were expressed as percentage of events ± SEM and evaluated by two-way ANOVA with Bonferroni post-test ∗p < 0.001; compared to negative control group; ∗∗p < 0.001 compared to supernatant A. (B–E). Dotplot graph showing the behavior and marking percentages of each group.

Except for the negative control group, where only culture medium was added, all BMDMs groups exposed to the four supernatants (A, B, C, and D) showed CD11b positivity above 50 %. This suggests that the supernatants induced polarization towards the M1 phenotype (Fig. 9). Supernatant A exhibited the lowest CD11b percentage at approximately 56 %, whereas supernatants B, C, and D showed progressively higher percentages. Supernatant D notably had a CD11b percentage above 81 %, comparable to BMDMs groups treated directly with LPS and 1,2,4-oxadiazole derivative 2.

**Fig. 10:**
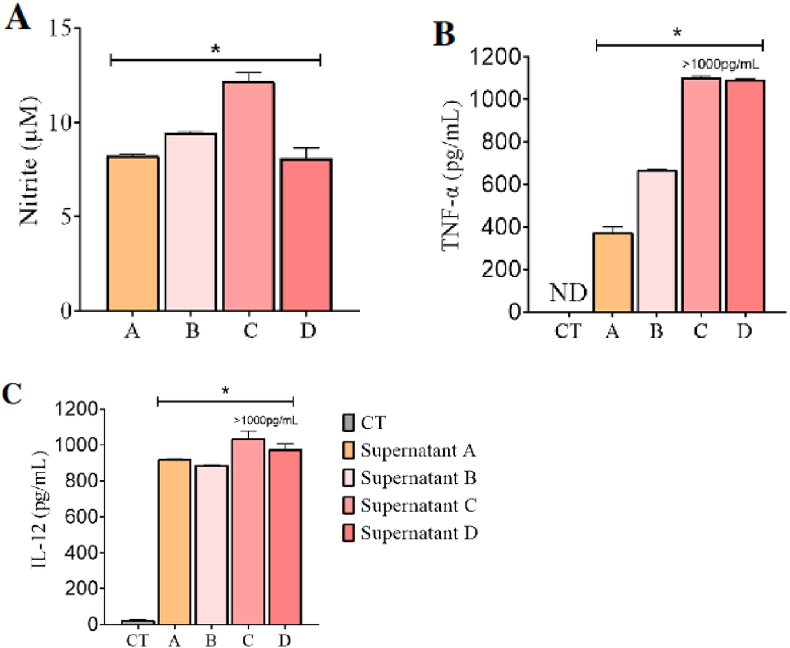
Dosage of the M1 markers in BMDMs supernatants after 48h of exposure to murine melanoma supernatants. (A, B and C) Column graph indicating concentration of nitrite, TNF-α and IL-12 respectively. Data were expressed as mean ± SEM and evaluated by one-way ANOVA with Dunnet's post-test ∗p < 0.001 compared CT. ND = Not detected.

Nitrite, TNF-α, and IL-12 assays results are shown in Fig. 10. Nitrites were detected in all groups with significant differences compared to the negative control. Group C had the highest concentration exceeding 10 μM. In all groups, TNF-α was detected at significantly higher levels compared to the negative control. Groups C and D exhibited values exceeding the detection range of the kit (>1000 pg/mL). These groups induced TNF-α production levels higher than those induced by LPS. IL-12 was also detected in all groups, with concentrations above 800 pg/mL and significant differences from the negative control. Group C showed values above the kit's detection range. Exposure with all four supernatant groups induced IL-12 production in greater amounts compared to LPS and 1,2,4-oxadiazole derivative 2.

### 3.6 1,2,4-Oxadiazole derivative 2 displays synergistic effect on the polarization induced by LPS and supernatants from murine melanoma

**Fig. 11:**
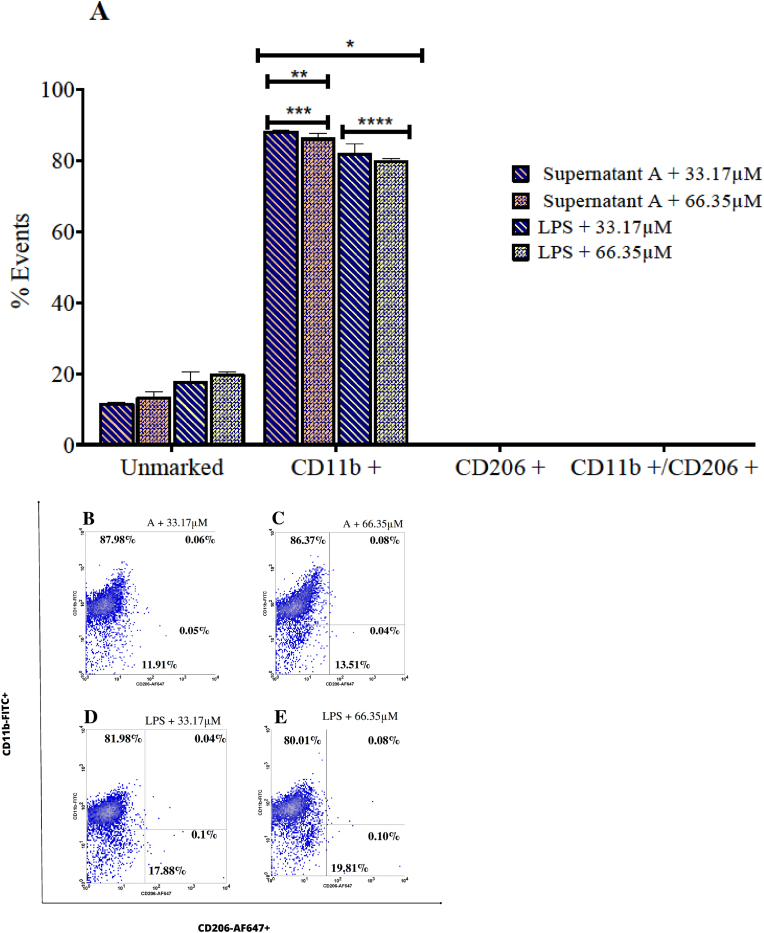
1,2,4-oxadiazole derivative 2 + murine melanoma sets and 1,2,4-oxadiazole derivative 2 + LPS sets polarized BMDMs to M1 phenotype after 48h exposure. (A) Column graph indicating the percentage of unmarked cells, CD11b+, CD206+ and CD11b+/CD206+. Unstimulated cells acted as a negative control (M0). Data were expressed as percentage of events ± SEM and evaluated by two-way ANOVA with Bonferroni post-test ∗p < 0.001; compared to negative control group; ∗∗p < 0.001 compared 1,2,4-oxadiazole derivative 2 + LPS sets; ∗∗∗p < 0.001 compared supernatant A; ∗∗∗∗p < 0.001 compared LPS. (B–E). Dotplot graph showing the behavior and marking percentages of each group.

Based on data from previous assays, we analyzed the impact of exposure to the 1,2,4-oxadiazole derivative 2 + Supernatant A and 1,2,4-oxadiazole derivative 2 + LPS sets on the phenotype of BMBMs. All data are presented in Fig. 11. All groups exhibited strong positivity to CD11b. 1,2,4-oxadiazole derivative 2 + Supernatant A sets showed approximately 88 % of positivity to CD11b, significantly higher than Supernatant A isolated (56 %). 1,2,4-oxadiazole derivative 2 + LPS sets showed approximately 82 % of positivity to CD11b, similar to LPS isolated (79 %). The groups exposed to 1,2,4-oxadiazole derivative 2 + Supernatant A showed higher CD11b positivity compared to the groups stimulated with 1,2,4-oxadiazole derivative 2 + LPS (%). 1,2,4-oxadiazole derivative 2 appears to have a synergistic effect with Supernatant A in polarizing towards the M1 phenotype.

**Fig. 12:**
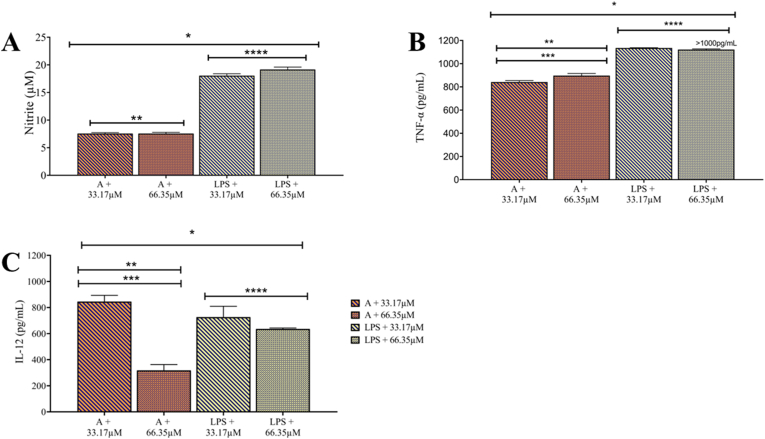
Dosage of the M1 markers in BMDMs supernatants after 48h of exposure 1,2,4-oxadiazole derivative 2 + murine melanoma and 1,2,4-oxadiazole derivative 2 + LPS sets. (A, B and C) Column graph indicating concentration of nitrite, TNF-α and IL-12 respectively. Data were expressed as mean ± SEM and evaluated by one-way ANOVA with Dunnet's post-test ∗p < 0.001 compared CT; ∗∗p < 0.001 compared 1,2,4-oxadiazole derivative 2 + LPS; ∗∗∗p < 0.001 compared supernatant A; ∗∗∗∗p < 0.001 compared LPS.

In the nitrite quantification assays, all groups showed significantly higher levels compared to the negative control group. Exposure to 1,2,4-oxadiazole derivative 2 + LPS sets increased nitrite concentration by more than three times compared to 1,2,4-oxadiazole derivative 2 + Supernatant A sets (Fig. 12A). When comparing 33.17 μM + LPS (18.03 μM) and 66.35 μM + LPS (19.15 μM) with LPS isolated (87.44 μM) a reduction in nitrite concentration by over four times was observed. Comparing 33.17 μM + Supernatant A and 66.35 μM + Supernatant A with Supernatant A isolated, no significant changes were observed in nitrite concentrations, which remained around 7 μM and 8 μM respectively.

In Fig. 12B, high TNF-α production was observed for all groups. The 1,2,4-oxadiazole derivative 2 + LPS sets exhibited TNF-α levels exceeding the detection limit of the kit, quantified as greater than 1000 pg/mL. Comparison with isolated LPS (806.30 pg/mL) suggests that 1,2,4-oxadiazole derivative 2 likely enhanced TNF-α production (>1000 pg/mL), resulting in higher levels detected in the combined stimulation compared to LPS alone. The same effect is observed when comparing 33.17 μM + Supernatant A (841.78 pg/mL) and 66.35 μM + Supernatant A (897.09 pg/mL) with Supernatant A isolated (373.74 pg/mL).

In all groups evaluated, IL-12 cytokine was detected, whereas it was absent in the negative control group. Within the 1,2,4-oxadiazole derivative 2 + LPS and 1,2,4-oxadiazole derivative 2 + Supernatant A exposure sets, significant differences were observed between the lowest and highest concentrations (Fig. 12C). Despite efforts to ensure accuracy in the ELISA assay for IL-12 detection, the high variation in detected values could be attributed to pre-analytical and analytical errors. Nevertheless, both sets showed different IL-12 levels compared to LPS isolated and Supernatant A isolated. Specifically, concentrations of IL-12 were higher in 33.17 μM + LPS (726.58 pg/mL) and 66.35 μM + LPS (635.95 pg/mL) compared to LPS isolated (585.48 pg/mL). Conversely, lower IL-12 levels were detected in 33.17 μM + Supernatant A (845.81 pg/mL) and 66.35 μM + Supernatant A (318.00 pg/mL) compared to Supernatant A alone (921.30 pg/mL).

## 4 Discussion

Melanoma skin cancer accounts for about 1 % of all skin cancers; however, it is the most aggressive and dangerous, responsible for 90 % of deaths from this type of pathology [31]. Additionally, one of the main problems associated with these types of tumors is their resistance to chemotherapeutic drugs and the high recurrence rate that these tumors can have after removal [26]. In this context, the search for alternative therapies is a viable option to improve the treatment of melanoma skin cancer and provide a better quality of life for patients [32].

The N-cyclohexyl-3-(3-methylphenyl)-1,2,4-oxadiazol-5-amine was obtained following the synthetic chemical route previously reported [22]. In cell viability assays, we found that 1,2,4-oxadiazole derivative 2 was able to inhibit the growth of murine melanoma cells compared to the control group (IC_50_ 50.99 μM), and of BMDMs compared to the control group (132.70 μM), demonstrating greater selectivity for the tumor cell line than BMDMs. Another notable finding is that the necrotic pathway was demonstrated as the mechanism of cell death for the B16–F10 cell line.

These results are in line with others that evaluated the activity of a range of synthetic indole 1,2,4-oxadiazole derivatives and found that two other compounds, 3 and 4 (Supplementary Fig. 13) [33], showed high cytotoxicity against the COLO 320 and MIA PaCa-2 cell lines at concentrations below 18 μM. Both were subjected to an evaluation of the cell death pathway by apoptosis and/or necrosis, and unlike 3, the 4 molecule caused plasma membrane rupture with the accumulation of cell debris in the supernatant, characterizing death by necrosis. In another study, the authors also evaluated the cell death pathway by apoptosis and/or necrosis induced by the 1,2,4-oxadiazole synthetic derivative compound 5f, it was found that increasing the concentration of the molecule caused a transition from early apoptosis to late apoptosis and necrosis. They also noted that this transition is dose-dependent and similar to that which occurs with well-established anticancer agents such as cisplatin, etoposide, and doxorubicin [34] and possibly with the 1,2,4-oxadiazole derivative 2 used in this present study. This antitumor activity may be related to the lipophilic nature of oxadiazoles and their greater membrane permeability. Additionally, their ability to form strong hydrogen bonds may trigger the irreversible inhibition of different enzymes essential for the development and replication of various cell types, including cancer [35].

Given the historical failure of monotherapy in treating advanced melanomas, the combined use of immunotherapy with IL-2, CTLA-4 inhibitors, adoptive T cell transfer (ATCT), and BRAF inhibitors (V600E) enhances immune responses and has become the most effective modality with very positive results for patients [36]. TAMs are the predominant leukocytes in solid tumors and tend to assume the M2 phenotype, which promotes tumor development, the growth of new blood vessels, and reduced immunosurveillance. These primary functions make these cells therapeutic targets to be explored. The main strategies are based on the elimination of cells already present in the TME, blocking the recruitment of new macrophages, and repolarizing the existing ones to the M1 phenotype [37].

Here, after the stages of bone marrow collection, CSF-M induced differentiation, and confirmation by flow cytometry and IC_50_ calculation, BMDMs were used in assays for phenotypic evaluation after stimulation with 1,2,4-oxadiazole derivative 2, LPS, and supernatants from murine melanoma. The experiment conducted with BMDMs is based on the hypothesis that the integrin CD11b plays an essential role in regulating macrophage polarization and is also important in the activation of NF-kB, which results in M1 macrophage that secretes NO and pro-inflammatory cytokines [38]. After monitoring the increase of CD11b in BMDMs treated with 1,2,4-oxadiazole derivative 2, we chose to culture the macrophages with the supernatants to demonstrate that the TME would not be sufficient to inhibit the commissioning promoted by the treatment and that the macrophages would maintain their inflammatory *in vitro* profile. Additionally, nitrite, TNF-α, and IL-12 levels were measured in these supernatants collected from the stimulation groups. The use of CSF-M in the differentiation process of macrophages from immature bone marrow cells is commonly employed in *in vitro* research. Mature cells exhibit high phagocytic capacity, low antigen-presenting capacity, and high expression of markers CD14, CD163, and CD206, which are more related to an immunosuppressive phenotype [39,40]. Although this factor may tend towards the formation of M2 macrophages, our results showed that the negative control predominantly did not induce phenotypic changes in the differentiated macrophages; thus, the observed changes were due to the applied stimuli.

The present results indicate that exposure to LPS showed significant polarization to M1, as well as elevated levels of TNF-α. These results were quite expected. LPS was assumed to be a positive control since it is a well-known and potent activator of macrophages via TLR4/NF-кB signaling, triggering the production of TNF-α, IL-1β, IL-6, IL-12, and NO, which positively feedback this M1 polarization pathway [41]. When compared with 1,2,4-oxadiazole derivative 2, we observed a slightly higher percentage of M1 polarization than with LPS, low levels of TNF-α, and undetectable amounts of nitrite and IL-12 (data not shown). The pathway by which 1,2,4-oxadiazole derivative 2 alters the macrophage phenotype does not appear to be the same as that of LPS. We also observed that BMDMs were polarized to M1 after contact with all groups of supernatants. The supernatants containing traces of 1,2,4-oxadiazole derivative 2 (Supernatants B, C, and D) were able to increase the percentage of M1 cells compared to Supernatant A, which did not contain any traces of 1,2,4-oxadiazole derivative 2. It was also possible to observe that the exposure caused an increase in the levels of nitrite, TNF-α, and IL-12.

Similar to the isolated exposures, 1,2,4-oxadiazole derivative 2 + Supernatant A sets and 1,2,4-oxadiazole derivative 2 + LPS sets were also able to modulate BMDMs, polarizing them to the M1 phenotype. We observed that the presence of 1,2,4-oxadiazole derivative 2 had a synergistic effect on M1 polarization, clearly evidenced by the considerable increase from 56 % to 88 % among BMDMs exposure with Supernatant A and 1,2,4-oxadiazole derivative 2 + Supernatant A sets. The same effect was identified in the quantification of TNF-α for both combinations compared to isolated exposure. For IL-12, we observed a synergistic effect for 1,2,4-oxadiazole derivative 2 + LPS sets compared to isolated stimulation. Regarding nitrite levels, a reduction was observed for 1,2,4-oxadiazole derivative 2 + LPS sets compared to isolated exposure. The exposure to supernatants from previous assays where 1,2,4-oxadiazole derivative 2 induced necrosis in murine melanoma cells aimed to replicate a methodology that simulates the influence of soluble products contained in the supernatants on the macrophage phenotype. In addition to unknown secreted products and tumor antigens, the supernatants contained variable amounts of DAMPs [42]. Toll-like and NOD-like receptors are distributed on the surface and inside macrophages. Once activated by DAMPs, they trigger polarization to the M1 phenotype and consequently an inflammatory response [43]. We believe that the activation of these receptors prevailed in the polarization to M1. We should also consider the increasing percentage of M1 cells as supernatants with higher concentrations of 1,2,4-oxadiazole derivative 2 were used in the original assay.

Immunogenic cell death (ICD) has gained relevance due to its ability to enhance both innate and adaptive immune responses. Treatments such as anthracyclines, cyclophosphamide, oxaliplatin, radiotherapy, virotherapy, and photodynamic therapy have shown robust increases in antitumoral immunity through ICD [44]. A common characteristic among ICD processes is the release of damage-associated molecular patterns (DAMPs). Calreticulin, heat shock proteins (HSPs), high mobility group box 1 protein (HMGB1), Adenosine triphosphate (ATP), DNA, and Ribonucleic acid (RNA) are examples of DAMPs [45].

Most TAMs originate from circulating monocytes. Factors derived from tumors (FDTs) are responsible for recruiting these circulating monocytes to the TME. C–C motif ligand (CCL) 2 has been identified as the primary recruiter, with CCL3, CCL4, CCL5, CXCL12, and CSF-M also playing roles. Once recruited, monocytes undergo transformation into macrophages and quickly tend to polarize towards the M2 phenotype [46]. TAMs M2 perform essential functions to support tumor growth. They produce VEGF and MMP9, which induce angiogenesis and lymphangiogenesis. IL-1β, IL-8, and TGF-β contribute to epithelial-mesenchymal transition. Cathepsins, Matrix metalloproteinase (MMP) 2, MMP7, and MMP9, along with serine proteases, degrade the extracellular matrix promoting invasiveness and metastasis. TAMs M2 express high levels of PD-L1, PD-L2, CD80, and CD86 molecules, which interact with receptors present on Cytotoxic T cells (CTLs), B lymphocytes, and natural killer (NK) cells, resulting in the inhibition of their activation. TGF-β and IL-10 promote the formation of Treg lymphocytes from CD4^+^ T lymphocytes, while increasing the expression of Foxp3 and CTLA-4 [47].

Research in cancer immunotherapy focused on TAMs as therapeutic targets is gaining importance. An approach that has shown promise is the repolarization of the M2 phenotype to M1. We know that the macrophage phenotype is determined by the stimuli received in the TME. Factors such as the concentration of agonists binding to their receptors, the duration of pathway activation post-stimulation, and redundancy in signaling pathways in case the main pathway fails, also influence this determination. Despite the complexity involved, the use of molecules designed to block signaling pathways that lead macrophages towards a pro-tumoral phenotype has proven effective in preclinical and clinical studies [48].

M1 macrophages can respond rapidly to stimuli and producing NO, which in turn can kill invading viruses and bacteria. It is also attributed to their ability to induce tumor cell death through the same mechanism. In addition to these roles, NO is also involved in the differentiation of T lymphocytes into the Th1 profile and the development of antibody-producing B lymphocytes [49]. Some studies have shown an increase in NO concentration due to the use of oxadiazole compounds [50,51]. The authors highlighted that the flexibility of this class allowed for the synthesis of structures containing ester nitrates that can easily release NO into the environment, thereby conferring antitumor properties. This parallel antitumor pathway, distinct from the one investigated in this study, underscores the potential of the oxadiazole class.

The increase in concentration of pro-inflammatory cytokines in the TME is directly related to enhanced cytotoxic activity of immune cells. TNF-α is a potent inflammatory mediator primarily produced and secreted by M1 macrophages. Its production triggers a variety of cellular responses including activation of pro-inflammatory gene transcription, secretion of cytokines and chemokines, activation of other macrophages, neutrophils, dendritic cells, NK cells, and CTLs, enhanced antigen presentation and phagocytosis, and can induce direct tumor cell death via activation of caspase-8. IL-12 is a pro-inflammatory cytokine primarily produced and expressed by antigen-presenting cell (APCs) such as dendritic cells and macrophages. In the context of the TME, it stimulates effector cells of both innate and adaptive immunity, leading to enhanced cytotoxic immune responses, polarization of macrophages towards M1, transformation of CD8^+^ T lymphocytes into CTLs, and recruitment of NK cells. IL-12-mediated activation triggers the release of Th1 cytokines like IFN-γ, resulting in inhibition of Treg lymphocytes and increased expression of MHC class I molecules on tumor cells facilitating recognition [52,53].

In their study on the anti-hepatitis B virus (HBV) activity of 1,2,4-oxadiazole derivatives [54], found that compound 11 acted as an agonist on TLR-8 receptor, activating the NF-κB pathway in peripheral blood mononuclear cells. They also demonstrated that binding to TLR8 led to dose-dependent expression of TNF-α and IL-12. Our results align with these findings, corroborating the immunomodulatory potential of 1,2,4-oxadiazole derivatives. Conversely, studies previously reported [34,50,[55], [56], [57], [58]] have shown opposite modulatory effects on the immune system, with reduction in pro-inflammatory cytokines. This study did not aim to specify the mechanisms by which 1,2,4-oxadiazole derivative 2 modulates BMDMs. As mentioned, the M1 phenotype is associated with activation of signaling pathways such as JNK, P38, NF-kB p65, and JAK/STAT1. The significant involvement in polarizing BMDMs towards the M1 phenotype suggests that the molecule N-cyclohexyl-3-(3-methylphenyl)-1,2,4-oxadiazol-5-amine participates in activation of one or more of these pathways. Some limitations of this present study include the lack of quantification of other important pro-inflammatory and anti-inflammatory cytokines and chemokines by employing other analytical assays, as well as identifying cellular markers involved in phagocytosis and antigen presentation processes.

In conclusion, 1,2,4-oxadiazole derivative 2 demonstrates cytotoxicity against the B16–F10 murine melanoma cell line by inducing cell death via necrosis. It also displays immunomodulatory profile by modulating *in vitro* macrophages towards the M1 phenotype despite M2, in addition to contributing to increased TNF-α and IL-12 levels. The results achieved highlight the importance of the 1,2,4-oxadiazole compound class and, specially, the significant potential of 1,2,4-oxadiazole derivative 2 to be explored as an immunotherapeutic agent against tumors.

## CRediT authorship contribution statement

**Héverton Mendes Araújo:** Writing – original draft, Methodology, Investigation, Formal analysis. **Gabriel Acácio de Moura:** Writing – original draft. **Yasmim Mendes Rocha:** Writing – original draft. **Cristian Vicson Pinheiro Gomes:** Writing – original draft. **Valentina Nascimento e Melo de Oliveira:** Resources, Methodology. **Ronaldo Nascimento de Oliveira:** Resources, Methodology. **Larissa Deadame de Figueiredo Nicolete:** Writing – original draft. **Emanuel Paula Magalhães:** Methodology, Investigation. **Ramon R.P.P.B. de Menezes:** Resources, Methodology. **Roberto Nicolete:** Writing – original draft, Supervision, Project administration, Investigation, Funding acquisition, Conceptualization.

## Declaration of competing interest

The authors declare that they have no known competing financial interests or personal relationships that could have appeared to influence the work reported in this paper.

## Data Availability

Data will be made available on request.

## Abbreviations

7-AAD: 7-Aminoactinomycin D
AxPE: Annexin V Conjugated with Fluorochrome Phycoerythrin
BMDMs: Bone Marrow-Derived Macrophages
COX: Cyclooxygenase
CTLA-4: Cytotoxic T-lymphocyte-associated protein 4
FDTs: Factors derived from tumors
FBS: Fetal bovine serum
FCS: Forward Scatter
IFN-γ: Interferon-gamma
LPS: Lipopolysaccharide
CSF-M: Macrophage Colony-Stimulating Factor
NED: N-(1-naphthyl) ethylenediamine Dihydrochloride
NO: Nitric oxide
NO2: Nitrite
PBS: Phosphate-buffered saline
PD1: Programmed cell death protein 1
ROS: Reactive oxygen species
RNS: Reactive nitrogen species
SSC: Side Scatter
TAMs: Tumor-associated macrophages
TNF-α: Tumor Necrosis Factor-alpha
TME: Tumor microenvironment
