# Supplementary figures and images for: In vitro antitumor and immunomodulatory activities of 1,2,4-oxadiazole derivatives
Source: Biochem Biophys Rep. 2025 Feb 10;41:101950. doi: 10.1016/j.bbrep.2025.101950 (PMC11868951; doi:10.1016/j.bbrep.2025.101950)

**
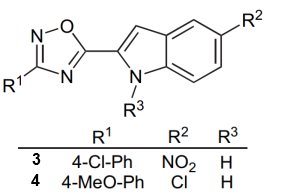
**

**Supplementary** **Figure 13**: 3 and 4 chemical structures

Supplement: Multimedia component 1 [file mmc1.docx]
